# The Role and Impact of Extracellular Vesicles in the Modulation and Delivery of Cytokines during Autoimmunity

**DOI:** 10.3390/ijms21197096

**Published:** 2020-09-26

**Authors:** Mohammed Tayab Hussain, Asif Jilani Iqbal, Lucy Victoria Norling

**Affiliations:** 1 William Harvey Research Institute, Barts and the London School of Medicine, Queen Mary University of London, London E1 4NS, UK; m.t.hussain@qmul.ac.uk; 2 The Institute of Cardiovascular Sciences, College of Medical and Dental Sciences, University of Birmingham, Birmingham B15 2TT, UK; a.j.iqbal@bham.ac.uk; 3 Centre for Inflammation and Therapeutic Innovation, Queen Mary University of London, London E1 4NS, UK

**Keywords:** extracellular vesicles, exosomes, inflammation, autoimmunity, cytokines

## Abstract

Cytokines and extracellular vesicles are two methods of initiating and maintaining cellular crosstalk. The role of cytokines in the initiation, progression, and resolution of inflammation has been well studied and more so, their pathophysiological role in the development of autoimmune disease. In recent years, the impact of extracellular vesicles on the progression of autoimmunity has become more widely appreciated. In this review, we discuss the mechanisms that allow extracellular vesicles of various sources to modulate cytokine production, and release, and how extracellular vesicles might be involved in the direct delivery and modulation of cytokine levels. Moreover, we explore what challenges are faced by current therapies and the promising future for extracellular vesicles as therapeutic agents in conditions driven by immune dysregulation.

## 1. Cytokines, Inflammation, and Autoimmunity 

Secreted, soluble factors such as hormones, growth factors, and cytokines are key drivers of cellular communication. Cytokines are small, soluble proteins weighing thirty kilodaltons or less that are synthesized and secreted by a range of cells, including both immune cells, such as neutrophils, B-, and T-cells, and stromal cells, such as endothelial cells and fibroblasts [1]. The effects of cytokines are pleiotropic in nature and any given mixture or single cytokine may result in functionally different but stereotyped outcomes in a context-dependent manner [2,3]. Their soluble nature enables cytokines to act in the local microenvironment or to also exert their influence in an endocrine manner. Local cytokine activity enables cells to self-regulate their expression and secretion with many feed-forward and negative feedback loops existing in most cytokine systems/hierarchies. Cytokine release is possible in any organ and compartment throughout the body and has far reaching effects on cell survival, differentiation, and activation [4]. Interestingly, cytokine interactions with their cognate receptors are high affinity and under physiological conditions occur at picomolar concentrations and as a result, their release, and consumption, remains highly regulated [5]. Nevertheless, the half-life of any given cytokine is relatively small, and degradation in extracellular fluids or blood occurs rapidly [6]. Cytokines are also able to act in a manner independent of secretion and can act as cell surface ligands. While soluble cytokines may promote unidirectional signalling into the target cells, cell surface cytokines can initiate bi-directional outside-in signalling [7].

The key physiological function of cytokines as a group of proteins is the initiation, maintenance, and resolution of inflammatory responses [8]. Inflammation is the stereotyped, non-specific response to conserved peptides, motifs, and signals, which may be pathogens or damage-associated molecular patterns. Under non-pathological conditions, inflammatory responses are typically self-limiting and immune cell recruitment and clearance is a tightly regulated and stepwise process [9]. The initial release of pro-inflammatory cytokines, such as tumour necrosis factor alpha (TNF-α) and interleukin 1 beta (IL-1β), by tissue-resident cells induces the expression of adhesion molecules, such as selectins and integrins, which facilitates immune cell recruitment [10]. While the function of most pro-inflammatory cytokines might be functionally redundant and overlapping, certain caveats exist. For example, TNF-α promotes adhesion molecule expression on both leukocytes and endothelial cells, while IL-1β predominantly affects endothelial cells [11]. Furthermore, while TNF-α-mediated immune cell recruitment can efficiently occur independent of certain junctional proteins, recruitment through the IL-1β stimulatory pathway is decreased [12]. TNF-α is also capable of directly activating and stabilizing a pro-inflammatory phenotype in a range of cells, such as neutrophils and monocytes, which in turn stimulates them to upregulate the expression of their own range of cytokines and proteins in a self-amplifying loop [13,14]. This cycle of primary activation and secondary cytokine release can be observed with most pro-inflammatory cytokines [15,16,17]. 

Interleukin-6 (IL-6) levels are elevated as a result of secondary secretion in response to both TNF-α and IL-1β, interestingly, IL-6 feeds back to stimulate further TNF-α and IL-1β secretion [18]. Zheng et al., amongst others, have shown this to be true as it is possible to significantly decrease circulating IL-6, while also ameliorating clinical symptoms, in immunologically challenged mice with global deletions in IL-1β [19]. Finally, while pro-inflammatory cytokines are important for the initiation and maintenance of inflammation the appropriate anti-inflammatory signals must be integrated into any given system to terminate inflammation and promote immune cell clearance and tissue regeneration. Anti-inflammatory cytokines, such as interlekin-10 (IL-10) are able to supress pro-inflammatory gene expression and skew the phenotypic switch of immune cells away from a pro-inflammatory profile towards one that favours resolution and regeneration [3]. A number of cytokines also exert differential effects depending on the environmental context in which they are active or indeed the mechanism through which they may act. IL-6 is one such pro-inflammatory cytokine, whereby classical signalling induces anti-inflammatory effects and trans-signalling induces inflammation [3,18].

When the activity of cytokines, such as TNF-α, IL-1β, and IL-6, becomes dysregulated it is understood to be key in the pathophysiology of autoimmune diseases [20]. Autoimmunity is a broad term encompassing a range of diseases characterized by the loss of central tolerance and the maintained, pro-inflammatory immune response directed at host antigens. From an aetiological perspective, most autoimmune diseases have been reported to be multi-factorial with genetic polymorphisms infectious and environmental factors playing a role in their pathophysiology. The full spectrum of autoimmune disorders spans organ-specific to systemic diseases and includes conditions such as type 1 diabetes (T1D), rheumatoid arthritis (RA), systemic lupus erythematosus (SLE), and multiple sclerosis (MS) [21]. The major autologous antigens driving these diseases have been summarized by others (reviewed by Suurmond et al. [22]).

TNF-α is implicated across a range of autoimmune conditions and diseases and has received great attention as a therapeutic target [23]. Organ-specific increases in TNF-α have been shown to induce innate immune cell infiltration and activation, tissue resident cell activation, and pro-inflammatory cytokine secretion [24,25,26]. In particular, the blockade of TNF-α has been shown to ameliorate the inflammatory symptoms of RA and attenuate the extent of joint and cartilage erosion [27]. Similarly, increases in serum IL-6 levels have been reported as biomarkers of systemic B-cell activation, and the extent of circulating IL-6 reflected radiographic RA progression [28]. IL-6 stimulation of B-cells induces their differentiation into plasma cells and is accompanied by elevated circulating immunoglobulin levels, especially relevant in the circulating immune-complexes critical to the progression of lupus [29,30]. IL-6, as well as IL-1β, can also induce innate immune cell recruitment, and inhibit the regulatory phenotype of CD4^+^ T-cells [31]. Levels of IL-1β have been found to be elevated in the blood, cerebrospinal fluid, and central nervous system lesions of MS patients [32]. Pro-inflammatory T-cells from patients with MS have been shown to stimulate IL-1β production from myeloid cells, which in turn drives the continued expansion of inflammatory T-cells [33]. Similar increases in systemic IL-1β have been observed in patients with RA, and the success of clinical trials for anti-IL-1β therapeutics demonstrates the significant and pathogenic role of IL-1β during autoimmunity. However, anti-IL-1β therapeutics, such as anakinra, have not managed to emulate similar success in the clinic following concerns surrounding cost and off-target effects [34]. Not only is the production of pro-inflammatory cytokines exaggerated in disease, but the activity of anti-inflammatory cytokines inhibiting them is also dysregulated. Interleukin-37 is a naturally occurring antagonist of IL-1 family member cytokines, its release by macrophages has been shown to inhibit pro-inflammatory cytokine release in mast cells, the major pathological cell type in SLE. Paradoxically, circulating IL-37 is increased in patients with SLE, and it is thought that this reflects an IL-1/IL-37 negative feedback loop in the context of mast cell insensitivity to IL-37 [35]. Nevertheless, although the role of soluble factors, such as cytokines, and physical cell–cell contact as mediators of cell communication and drivers of autoimmunity are well described, extracellular vesicles (EVs) have remained largely underappreciated, that is, until recently [36].

## 2. Extracellular Vesicles: What, Where, and How? 

EVs were initially discovered as platelet-derived particles with prothrombotic properties and aptly named “platelet dust”. However, it is now understood that EVs can originate from virtually all cell type, including stromal and immunological sources [37,38]. Cell–cell communication via EVs denotes an evolutionarily conserved mechanism to enable the transmission of nucleic acids, lipid mediators, and protein cargo, such as cytokines [39]. The cargo of EVs is primarily determined by the cellular source of origin and the activation status or phenotype of the parent cell in question [40]. Therefore, the cargo of EVs derived from a cell with a wholly anti-inflammatory profile would on the whole also be anti-inflammatory, similarly, pro-inflammatory EV cargo would reflect a wholly pro-inflammatory cell of origin. Owing to the complexity created by differences in cargo, cellular source, and importantly, particle size, EVs are heterogenous in nature [41]. 

Based on particle size, EVs can be organized into three main groups; however, additional subgroups also exist, dependent on annexin-1 or arrestin-domain-containing protein 1 content, for example, but are not discussed herein [42]. EVs described as exosomes can be observed in the 30–100 nanometre (nm) diameter range [43]. Exosomes are generated by the inward budding of the endosomal membrane in the luminal space of multivesicular endosomes undergoing maturation. Fusion of these endosomes to the cell surface leads to the release of exosomes [44,45]. Notably, not all intraluminal vesicles are destined to be released as exosomes, as some multivesicular bodies may fuse with lysosomes and undergo destruction [46]. This process can occur in endosomal sorting complexes required for transport (ESCRT) either in a dependent or independent manner, the latter being dependent on ceramide and to an extent, tetraspanin expression [47]. EVs described as microvesicles are larger in diameter (150–1000 nm) and are synthesized in response to intracellular changes in calcium [48]. Increased intracellular calcium triggers a cascade of enzymes and G-protein-coupled receptors, which triggers phosphatidyl serine flipping from inside the cell to the outside, cellular depolarization, and cytoskeletal contraction [49,50]. Collectively, these processes result in the outwards blebbing and pinching of the phospholipid bilayer releasing microvesicles into the extracellular space [51]. The disruption of membrane asymmetry is considered an important step in microvesicle generation, but there is also growing evidence that microvesicle production can occur despite membrane asymmetry being maintained [52]. Finally, apoptotic bodies are the third group of EVs and are generated by membrane budding that occurs during apoptosis resulting in vesicles ranging from 1 to 5 micrometre (µm) in diameter [53].

The encapsulation of EV cargo by a lipid membrane allows the delivery of cargo to sites far from the cells of origin as the cargo is offered protection from destruction and enzymatic degradation [54]. However, whether EV cargo is packaged selectively, or not, remains a point of discussion. Currently, it is believed that while some cargo is indeed not packaged selectively, offering an overview of cellular content, there is also evidence for selective cargo packaging [55]. Margolis et al. have reported the heterogeneity of tumour-derived EVs based on density. It was shown that low-density EVs were not enriched for any specific microRNA (miR), based on size, it was suggested these were generated using the microvesicle biogenesis pathway. Interestingly, size analysis of high-density vesicles suggested they were generated by the exosomal biogenesis pathway and that these high-density exosomes were enriched specifically for miR-122. Further experimentation revealed that miR-122 was selectively packaged into these EVs by association with La, an RNA binding protein found to be upregulated in some cancers [56,57]. In the context of tumourigenesis and survival, it is thought this mechanism of selective packaging is responsible for the expulsion of anti-oncogenic microRNAs, such as miR-122, promoting tumour growth and metastasis [57]. Similar findings have also been reported for miR-100 and miR-23b in the context of tumour survival [58,59]. While these findings support the presence of mechanisms that enable both selective and non-selective cargo packing in EVs, it is currently unknown how this may translate into the context of autoimmunity and, indeed, non-miR EV cargo, such as cytokines. 

**Figure 1:**
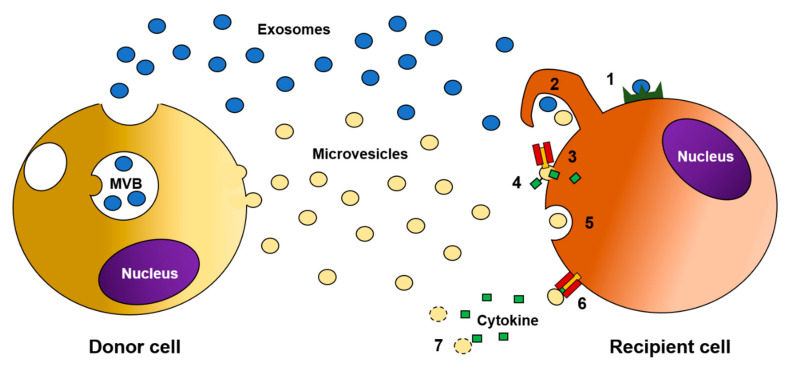
Mechanisms of cytokine delivery via extracellular release and uptake. Following extracellular vesicle (EV) release, through their respective biogenesis pathways, EVs can facilitate the intracellular delivery of cytokines into recipient cells by clathrin-mediated endocytosis (1), macropinocytosis (2), cell membrane fusion (3), and phagocytosis (4). EV membrane fusion also allows the lateral transfer of EV-tethered cytokines and cytokine receptors to the surface of recipient cells (5). Additionally, EV-tethered cytokines can directly interact with their cognate cell surface receptors on target cells (6). Finally, cytokines can be released from EV encapsulation and directly into the extracellular space whereby the cytokines in question can exert their effect (7) [60].

Once EVs are generated and shed, their cargo, such as cytokines, can signal into the extracellular environment via various routes (Figure 1) [60]. By measuring RNA copy numbers in any target cell, it is possible to detect the acquisition of microRNA-bearing EVs in any given cell population [61,62]. More direct observations of EV uptake, by immune cells specifically, have been made by the likes of Hyenne et al. In an elegantly designed study using zebrafish, it was possible to visualize the scavenging and consumption of tumour-derived EVs by macrophages using arm-like protrusions [63]. Further study is required to understand the mechanisms that directly govern EV homing and recruitment, but the repertoire of surface markers imbued by the cell of origin are undoubtedly important. There is a great deal of evidence that implicates EVs in the regulation of homeostatic processes, which includes but is not limited to, roles in coagulation [64], stem cell renewal and expansion [65], and inflammation [66]. However, as with most mediators of cellular communications, the relationship between EVs and inflammation is also intimately linked with the pathogenic role of EVs during autoimmunity [67].

## 3. The Role of EV Cargo in Modulating Cytokine Production during Autoimmunity

At present there are number of reports that have shown an increase in circulating EV levels during autoimmune conditions [68,69]. Knijff-Dutmer et al. demonstrated that circulating levels of platelet-derived microparticles were significantly elevated in RA patients compared to those in healthy volunteers, and the relative abundance of these EVs correlated directly to disease activity [70]. In another study, Berckmans et al. reported that the number of platelet- and leukocyte-derived EVs was also elevated, specifically in the synovium of RA patients [71]. Others have shown similar systemic increases in EV levels during SLE, Sjogren’s syndrome, and MS [72,73]. Viñuela-Berni et al. also demonstrated an increase in circulating EVs in SLE and RA and that these EVs could potently stimulate the release of interleukin 17 (IL-17), TNF-α, and interleukin 1 (IL-1) in vitro [74]. Interestingly, a paradoxical decease in circulating microvesicles was observed by Sellam et al. in the most severe examples of autoimmune disease [75]. It is thought that this is likely due to increased phospholipase A2 secretion and activity, which amplifies EV uptake as seen by Duchez and colleagues in neutrophils [75,76]. Others have used preclinical models to show the pathophysiological role EVs play in autoimmune disease. For example, Kavian et al. used a murine model of systemic sclerosis in order to demonstrate that chemically inhibiting microvesicle release with pantethine decreased the extent of skin and lung fibrosis compared to mice that had not received the inhibitor of EV release [77]. In a similar fashion, Deng et al. have shown the benefit of inhibiting the release of receptor activator of nuclear factor kappa-Β ligand (RANK-L)-positive EVs from osteoblasts in ovariectomized mice as a method of significantly reducing bone loss [78]. While the ovariectomized mouse does not represent a model of autoimmunity, the activity of RANK-L-positive EVs in the formation of mature, multi-nucleated osteoclasts, and indeed exaggerated bone loss, remains relevant to the study of RA [79,80].

EV cargo has been shown to modulate cytokine release during autoimmunity in a variety of ways [81]. For certain cell types, the requirement of specific EVs are reported as essential for cell maturation. Plasmacytoid dendritic cell maturation was shown to be regulated in response to EVs, which induced IL-6, IL-8, and co-stimulatory molecule expression from mature cells. Additionally, these EV-stimulated plasmacytoid dendritic cells were observed to initiate proliferation of allogenic naïve CD4^+^ T-cells and trigger their secretion of TNF-α and interferon gamma (IFN-γ). Importantly, the authors showed that EV-dependent maturation in this setting was specific to endothelial EVs and the effect was not reproduced by EVs from either platelets or lymphocytes [82].

The release of pro-inflammatory cytokines, such as TNF-α, interleukin 2 (IL-2), and IFN-γ, following antigen presentation and T-cell receptor activation, has been previously well documented [83]. Antigen-presenting cells (APCs) such as dendritic cells have alternatively been shown to take part in a process termed major histocompatibility complex (MHC) cross-dressing. MHC molecules enable the presentation of antigen to T-cell antigen receptors, importantly, MHC I receptors specifically interact with CD4^+^ T-cells and MHC II with CD8^+^ T-cells. MHC cross-dressing involves the decoration of APCs with EVs expressing MHC I and II loaded with antigen, or autoantigen, onto the cell surface at a high enough concentration and in the correct spatial orientation to efficiently trigger cognate T-cell receptor activation [84]. Even in the absence of a traditional APC, it is understood that EVs are able to elicit an efficient T-cell response [85]. The direct binding of EVs to T-cells has been previously described, as well as the expression of co-stimulatory molecules and antigen-loaded MHC on the surface of EVs [86,87]. In the context of autoimmunity, Skriner et al. have previously shown the association of citrullinated proteins with synovial exosomes from individuals with RA [88]. Others have shown that synovial macrophage exosomes isolated from patients with juvenile idiopathic arthritis specifically contain the nuclear oncoprotein, and autoantigen, DEK which stimulates joint inflammation [89]. Similarly, Hasilo et al. have reported the presence of diabetes autoantigens, such as glutamate decarboxylase and glucose transporter 2, in exosomes isolated from pancreatic islet cells [90].

Citrullinated proteins are a widely accepted biomarker of autoimmune disease, these are proteins in which arginine residues have been converted into citrulline. Citrullinated proteins are considered damage-associated molecular patterns and can trigger pro-inflammatory activity. Interestingly, others have shown that purification of synovial exosomes reveals the presence of citrullinated proteins, including vimentin, fibrinogen, and cluster of differentiation 5 antigen-like protein receptor, and that these extracellular vesicles are in association with immunoglobulin M (IgM) and immunoglobulin G (IgG) leading to the formation of immune complexes [88,91]. Microvesicle-immune complexes have been shown to potently induce a pro-inflammatory signature and chemotactic lipid mediator production, such as leukotrienes, from neutrophils in RA [91]. Immune complex formation is one of the hallmarks of SLE as the disease is characterized by the improper clearance of intracellular and nuclear proteins following cell death. Microvesicles bearing IgG can also be found in the serum of patients with SLE [92]. These EV immune complexes can trigger systemic complement activation and the release of neutrophil extracellular traps, a form of programmed cell death characterized by the outward expulsion of decondensed chromatin and granular contents. As a result, EV-immune complexes can induce the so-called interferon signature, which is typically observed in peripheral blood mononuclear cells from patients with SLE [93]. Moreover, EV-immune complexes can not only trigger inflammation in this capacity but are also essential in its maintenance and propagation. EV-immune complexes have also been shown to act on dendritic cells and, in doing so, induce the release of B-cell activating factor (BAFF). Dendritic cell release of BAFF in this way has been shown by Kang and colleagues to optimally drive the formation of B-cell memory [94].

While some dispute whether microRNAs associated with EVs can be found at concentrations high enough to induce functional changes, the vast majority believe otherwise [95,96,97]. In fact, there are a number of studies that explore the in vivo effect certain microRNAs can have in the context of cancer biology and importantly, autoimmunity [98,99]. For example, the lateral transmission of T-cell-derived EVs can induce pancreatic β cell apoptosis and C–C motif chemokine ligand 2, C–C motif chemokine ligand 7, and C–X–C motif chemokine ligand 10 release. Using a mouse model of T1D, the authors observed these T-cell EVs contained miR-142-3p, miR-142-5p, and miR-155, and the inhibition of these microRNAs protected recipient mice from developing diabetes [100]. Regulatory T-cells are known to attenuate inflammation, in part, through the secretion of anti-inflammatory cytokines such as IL-10; however, the relative abundance of these cells, and therefore, their anti-inflammatory secretome is decreased during autoimmunity. Exosomes found circulating in patients with multiple sclerosis have been shown to inhibit the differentiation of naïve CD4^+^ T-cells into regulatory T-cells by the delivery of the microRNA let-7i [101]. Similar to immune complexes, microRNA-containing exosomes also contribute to the interferon signature of SLE. Salvi et al. demonstrated that exosomal microRNAs rich in guanosine and uridine can drive plasmacytoid dendritic cell activation and maturation by triggering toll-like receptor 7 and, as a result, induce the secretion of TNF-α and IL-6 [102]. Despite the understanding of the individual roles that both EVs and cytokines exert during autoimmunity, the impact that cytokines associated with EVs may have in disease states remains widely underappreciated.

## 4. The Role of EVs in Delivering Cytokines

Immune cells can constitutively release cytokines in order to mediate homeostatic functions [103]. While it is understood that homeostatic cytokine release can occur through specific pathways of exocytosis, many have also shown that EVs are also utilized as tools for cytokine release/secretion [104]. Using a range of conditioned culture media, tissue explants, and bodily fluids, Fitzgerald et al. have shown a spectrum of heterogenous cytokine secretions in either free-soluble or EV-associated forms. Rather than EV association, or not, being a characteristic of any given cytokine, the authors have shown that, of the 33 cytokines assayed, the proportion of EV-associated versus free, soluble cytokine was dependent on the system of origin. For example, of the range of cytokines explored, the majority were found in their soluble form in placental villous explants. In contrast, the conditioned media from isolated T-cells and monocytes were found to contain the same panel of cytokines in, mostly, an EV-associated form (encapsulated or surface tethered). While on the whole, all cytokines were associated with EVs to some extent but eleven, in particular: IL-2, IL-4, IL-12p70, IL-17, IL-21, IL-22, IL-33, IFN-γ, C-X-C motif chemokine 11, transforming growth factor beta, and TNF-α, were observed to be associated with EVs more often when compared to the amount in a soluble form. The authors also found that activation altered the distribution of cytokines between soluble and EV-associated, to the extent where different pro-inflammatory stimuli, such as lipopolysaccharide (LPS) or polyinosinic:polycytidylic acid, induced differential profiles of cytokine distribution. Interestingly, different stimuli also altered whether the range of cytokines were either EV encapsulated or surface bound. The authors have demonstrated that EV-associated cytokines maintained their functionality in reporter cell lines; however, how free, EV-encapsulated, or EV-tethered cytokines may differ functionally remains to be seen [105].

Despite demonstrating biological activity, an important question must be considered, are circulating EV-associated cytokines at levels that might be physiologically, and clinically, significant? Fitzgerald et al. have reported that levels of free IL-1β and TNF-α in plasma from healthy donors were found at 7.5 and 4.9 picogram per millilitre (pg/mL), respectively. The proportion of EV-associated IL-1β and TNF-α was observed to be comparable to the amount free, 5.5 and 6.5 pg/mL, respectively [105]. Im et al. have shown that in serum from healthy young individuals approximately 30 pg/mL of TNF-α is associated with exosomes, and the proportion found in exosomes increases 3-fold with age, one of the largest risk factors associated with autoimmunity [106]. While the specific distribution during autoimmunity is not known, in vitro experimentation can begin to shine a light on how they might be related. Cytokine-stimulated T-cells have been shown to release IL-1β and TNF-α in EV-associated forms at average concentrations of 2.7 and 1.6 pg/mL, respectively, and only 0.1 pg/mL of both cytokines was found to exist in free form [105]. The current evidence therefore suggests that cytokines exist in an EV-associated form at significant levels. However, studies comparing the level of free, soluble cytokines and EV-associated ones during autoimmunity are required. Due to EV heterogeneity, it is now understood that the levels of certain cytokines, such as monocyte chemoattractant protein-1 (MCP-1), may vary between EVs of different sizes. Using a pancreatic β-islet cell line treated with a cocktail of cytokines, Giri et al. showed that the largest EV type, apoptotic bodies, had the greatest amount of MCP-1 associated with them (358 femtogram/7.6 × 10^4^ particles/1 × 10^6^ cells), while medium-sized microvesicles were associated with lower amounts (127.5 femtogram/8.25 × 10^5^ particles/1 × 10^6^ cells) and small extracellular vesicles were associated with the least (16.4 femtogram/9.1 × 10^7^ particles/1 × 10^6^ cells). The authors also demonstrated a positive relationship between cytokine concentration and EV size for IFN-γ, TNF-α, and IL-1β [107]. However, further investigation is warranted on whether this data reflects a paradigm whereby greater particle size facilitates greater payload levels or a potential mechanism for selective cytokine packaging based on EV size.

Currently, one of the most well-studied cytokines in relation to its association with EVs is IL-1β. On average, across several biological systems, the abundance of IL-1β has been shown to be equally distributed between EVs and free, soluble levels [105]. Unlike most other cytokines, IL-1β lacks a signal sequence and therefore has a non-conventional secretion pathway in association with EV release [108]. Previously, IL-1β has been shown to be released associated with exosomes from dendritic cells in patients with lupus [109]. IL-1β synthesis and release via EVs is highly regulated and dependent on the activation of the NOD-like receptor family pyrin domain containing 3 inflammasome. The inflammasome is a multiprotein complex, which directs inflammatory signalling in a range of cells and its activity has been shown to be a key driver in a range of autoimmune conditions, including RA and T1D [110,111]. It has been demonstrated that non-classical secretion of IL-1β is mediated by microvesicle shedding in monocytes, macrophages, dendritic cells, and microglia and following the activation of purinergic receptors on the surface of EVs, IL-1β is released into the extracellular space [112,113,114]. Increased purinergic receptor expression and signalling has been reported in the inflamed synovial tissue of arthritic rats and has been implicated in the pathogenesis of SLE [115,116]. Activation of synovial fibroblasts with IL-1β induces an arthritic phenotype, increasing cartilage degrading enzymes as well as IL-6 and vascular endothelial growth factor [117]. The pathogenic role of helper T-cells (Th cells) in autoimmunity has been well described; however in recent years, the role and impact of IL-17-secreting T-cells during autoimmune conditions such as RA, psoriasis, and SLE have been reported [118]. Hebel et al. have shown that IL-1β activates CD4^+^ T-cells, in conjunction with CD3 and CD28 stimulation, causing the release of IL-17. Sustained IL-1β signalling in combination with TGF-β and/or IL-6 causes committal of T-cell differentiation into a Th-17 fate [119]. Interestingly, the authors showed that IL-1β stimulation also induced the release of IFN-γ, and others have shown that IFN-γ induces the increased shedding of EVs by increasing the activity of EV-packaging machinery, such as interferon-stimulated gene 15 [119,120]. Ultimately, IFN-γ stimulates further inflammasome activation, therefore inducing further IL-1β synthesis and release via EVs in chronic inflammation. Stimulation by IL-1 family members has been seen to induce the release of IL-6-containing EVs from mast cells in a manner independent of de-granulation [121]. While the involvement of mast cells during autoimmunity is debated by some, data exists to support their pathogenic role in RA and multiple sclerosis [122]. In recent years, an autoimmune component has been implicated in the pathogenesis of amyotrophic lateral sclerosis and the release of EVs containing IL-6 from astrocytes is thought to contribute to disease pathogenesis and activity [123,124].

Systemic levels of IL-6, in part regulated by the packaging and secretion of microvesicles, have been shown to be increased in a range of autoimmune conditions [125]. Interestingly, multiple mechanisms of IL-6 signalling exist, these include traditional ligand–receptor interactions, receptor trans-signalling, and finally, IL-6/IL-6 receptor trans-presentation [126]. Moreover, IL-6 receptor expression has been reported on the surface of EVs [127]. Arnold et al. demonstrated that EV-bound IL-6 receptors can be donated to cells lacking receptor expression by vesicular fusion. This process of IL-6 receptor transmission has been termed joint reconstituted signalling and, inherently, increases the bioactivity of circulating IL-6 on a greater range of cells [128]. Evidence exists for the relevance of IL-6 signalling in inducing T-cell homing to pancreatic islets during T1D. However, this was not related to any increases in T-cell IL-6 levels, as observed by mRNA levels, but the increased expression of IL-6 receptor on the surface of T-cells [129]. Indeed, increased IL-6 signalling in T-cells has been shown to contribute to T-cell differentiation into IL-17-secreting cells and resistance to regulatory T-cell differentiation and their effector functions [130]. More recently, a role for the gut microbiota and changes in systemic LPS levels were found to drive the pathogenesis of autoimmune conditions [131]. To this end, Obregon et al. have shown that LPS-stimulated dendritic cells undergo exosome release, and these exosomes contain TNF-α, as well as, MHC-II, CD40, and CD83 [132]. Others have shown that exosomal-tethered TNF-α derived from mature dendritic cells induces endothelial inflammation [133]. Interestingly, Zhang et al. have shown that exosomes with a surface-tethered form of TNF-α, isolated from the synovial fibroblasts of RA patients, were also able to stimulate T-cells and in doing so, made them resistant to activation-induced cell death [134]. Liu et al. have reported similar effects of TNF-α associated exosomes on T-cells in Crohn’s disease [135]. The current evidence suggests that EVs make robust vessels for the delivery of cytokines, at levels that are clinically releavant during autoimmity. Therefore, is it possible to modulate EV activity in order to develop a therapeutic tool for treating autoimmunity?

## 5. Therapeutic Potential of Manipulating EVs 

Since the advent of monoclonal antibody therapies, the treatment of autoimmune conditions has come a long way. However, due to the heterogenous nature of most autoimmune conditions, monoclonal antibody therapies are not the silver bullet that they were once considered to be. The most widely used range of these antibody therapies targeting TNF-α have been observed to have varying degrees of success, with 25% to 38% and 21% to 42% of patients not responding to etanercept and infliximab monotherapies, respectively [136]. Naturally, these challenges may in part be explained by the additional layer of complexity added to the modulation of cytokine release via EVs. Fitzgerald et al. reported that cytokines packaged into EVs were not detectable by standard cytokine assays as the cytokines in question were shielded from antibody binding [105]. Moreover, others have shown that soluble TNF receptors are able to form homotrimers and, in doing so, act as a slow-release reservoir of cytokines when levels are low [137]. Interestingly, the expression of full-length TNF receptors have also been observed on the surface of EVs [138]; the biological significance remains to be determined but could suggest a mechanism for receptor transfer to recipient cells with poor expression or could act as an endogenous mechanism for counter-regulating inflammation by the release of decoy receptors. Indeed, Duong et al. have engineered exosomes to express human TNF receptors on their surface to act as decoys to mop up TNF-α from inflammatory environments and antagonize inflammatory signals [139]. Thus, despite the predominant pathogenic role of EVs during autoimmune conditions, many researchers are now attempting to manipulate these bioparticles towards a therapeutic end.

Interestingly, it has been reported that pregnant women with multiple sclerosis experience a reduction in disease activity and, the further on the pregnancy progresses, the greater this attenuation in clinical symptoms. Langer-Gould et al. attributed this effect towards a serum-derived factor and others have now identified these as exosomes released from placental tissue [140,141]. Indeed, placental villi remain an abundant source of cytokine-associated EVs, as shown by Fitzgerald et al. [105]. Exosomes derived from IL-10-stimulated dendritic cells have been observed to suppress collagen-induced arthritis in mice and reduce the clinical severity of established arthritis [142]. Similarly, others have genetically modified dendritic cells to express IL-4 or Fas ligand, a tumour necrosis family member, which triggers apoptosis, and used the resulting exosomes in order to attenuate delayed-type hypersensitivity and collagen-induced arthritis in mice [143,144]. 

**Figure 2:**
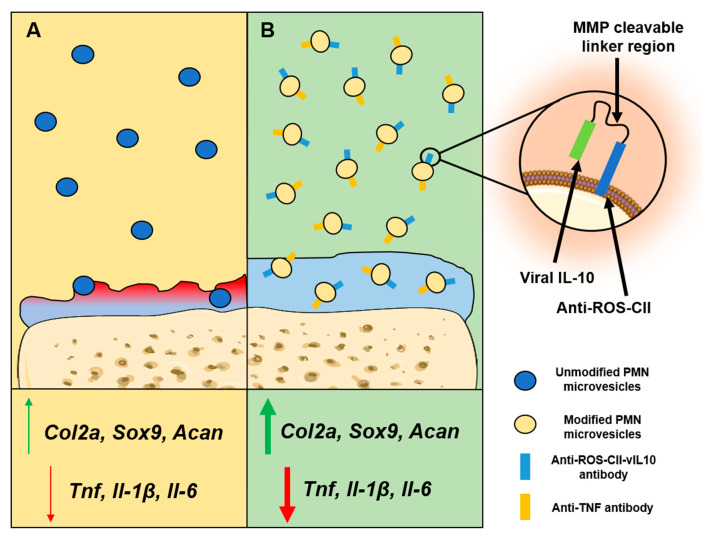
Therapeutic effects of polymorphonuclear-cell-derived extracellular vesicles. (**A**) Unmodified polymorphonuclear-cell-derived extracellular vesicles (PMN-EVs) administered to collagen-induced arthritis (CIA) mice are reported to exert anti-arthritic effects. PMN-EVs have been observed to penetrate cartilage where they can interact directly with chondrocytes. Upon cartilage infiltration, unmodified PMN-EVs inhibit the expression of genes responsible for pro-inflammatory cytokine production (*Tnf*, *Il-1β*, *Il-6*) and induce genes associated with cartilage anabolism (*Col2a*, *Sox9*, *Acan*). However, because such EVs lack any specific tools for homing to the injured joint, their effects are limited. (**B**) PMN-EVs modified to express antibodies targeting damaged type II collagen (anti-ROS-CII), found in arthritic but not healthy joints, are engineered to traffic into arthritic joints specifically. Using a variant of anti-ROS-CII, bound to viral IL-10 via a matrix metalloproteinase cleavable linker region (anti-ROS-CII-vIL-10), EVs were able to release an anti-inflammatory payload upon arrival to the injured joint. PMN-EVs co-engineered to express both anti-ROS-CII-vIL-10 and anti-TNF antibodies were shown to greatly reduce pro-inflammatory cytokine gene expression and enhance cartilage anabolism; to the extent whereby CIA joints treated with modified PMN-EVs were similar to healthy joints based on gross architectural appearance [151,154].

In recent years, the administration of mesenchymal stem cells (MSCs) has been investigated as a potential therapeutic approach for treating autoimmune diseases [145]. However, the use of MSCs provides challenges as they have shown to poorly meet clinical endpoints due to issues regarding clearance kinetics, homing, and biodistribution [146]. An alternative approach is to utilize exosomes derived from MSCs, these have been shown to induce potent anti-inflammatory effects and are able to attenuate a range of autoimmune conditions [147]. MSC-derived exosomes have been shown to delay the onset of T1D and experimental autoimmune uveoretinitis by suppressing the activation and development of Th1 and Th17 cells [148]. Similarly, the administration of MSC-derived exosomes in a model of collagen-induced arthritis was shown to inhibit T-cell proliferation in a dose-dependent manner and reduce the percentage of mature B- and T-cell subsets by inducing TGF-β and IL-10 production in target cells [149]. In essence, it is possible to engineer EVs with a range of anti-inflammatory cargoes, by either overexpressing cargo in source cells or by loading EVs with synthetic drugs or molecules [150]. With the addition of a range of EV surface receptors, such as immunoglobulins, lectins, MHC receptors, and viral proteins, it is possible to promote the selective recruitment of EVs to a specific tissue, site of injury, or cell population [125]. In fact, work from our group has demonstrated that neutrophil-derived EVs, which display anti-inflammatory activity, can be loaded with therapeutic cargo including anti-TNF-α as well as IL-10 and targeted to arthritic joints using cartilage specific antibodies to improve experimental arthritis (Figure 2) [151,152,153].

While the therapeutic potential of EVs is evident, more work is required to understand the precise mechanism governing cytokine packaging and release by microvesicles. The discovery of RNA-binding proteins in association with RNA found packaged into EVs raises the question, how might EV-associated cytokines differ from their free, soluble form. More importantly, how might EV association affect the terminal function of any given cytokine? The impact of shuttling EV-associated cytokines from the surface to an encapsulated form on biological function also requires further investigation. However, there is a clear role for EV-associated cytokines in the pathogenesis of autoimmune diseases, and these very same EVs may represent the future for treating the conditions they contribute to.
